# Experimental Investigation of Material Transfer on Bearings for Total Hip Arthroplasty—A Retrieval Study on Ceramic and Metallic Femoral Heads

**DOI:** 10.3390/jcm11143946

**Published:** 2022-07-07

**Authors:** Jessica Hembus, Lisa Rößler, Armin Springer, Marcus Frank, Annett Klinder, Rainer Bader, Carmen Zietz, Andreas Enz

**Affiliations:** 1 Biomechanics and Implant Technology Research Laboratory, Department of Orthopedics, Rostock University Medical Center, Doberaner Str. 142, 18057 Rostock, Germany; lisa.roessler@uni-rostock.de (L.R.); annett.klinder@med.uni-rostock.de (A.K.); rainer.bader@med.uni-rostock.de (R.B.); carmen.zietz@innoproof.de (C.Z.); 2 Medical Biology and Electron Microscopy Center, Rostock University Medical Center, Strempelstrasse 14, 18057 Rostock, Germany; armin.springer@med.uni-rostock.de (A.S.); marcus.frank@med.uni-rostock.de (M.F.); 3 Orthopedic Clinic and Policlinic, Rostock University Medical Center, Doberaner Str. 142, 18057 Rostock, Germany; andreaseugen.enz@med.uni-rostock.de

**Keywords:** material transfer, total hip arthroplasty, deposition pattern, femoral head

## Abstract

Metallic deposition is a commonly observed phenomenon on the surface of revised femoral heads in total hip arthroplasty and can lead to increased wear due to third bodies. In order to find out the origin and composition of the transfer material, 98 retrieved femoral heads of different materials were examined with regard to the cause of revision, localization, pattern and composition of the transfer material by energy dispersive X-ray spectroscopy. We found that in 53.1%, the deposition was mostly in the region of the equator and the adjacent pole of the femoral heads. The most common cause for revision of heads with metallic deposition was polyethylene wear (43.9%). Random stripes (44.9%), random patches (41.8%) and solid patches (35.7%) were most prevalent on retrieved femoral heads. Random patches were a typical pattern in ceramic-on-ceramic bearing couples. The solid patch frequently occurred in association with dislocation of the femoral head (55%). The elemental analysis of the depositions showed a variety of different materials. In most cases, titanium was an element of the transferred material (76.5%). In addition to metallic components, several non-metallic components were also detected, such as carbon (49%) or sulfur (4.1%). Many of the determined elements could be assigned with regard to their origin with the help of the associated revision cause. Since the depositions lead to an introduction of third-body particles and thus to increased wear, the depositions on the bearing surfaces should be avoided in any case.

## 1. Introduction

Nowadays, aseptic loosening due to wear particles is still the main cause for total hip revisions [1,2]. Numerous retrieval studies reported dark shimmering areas on the surface of explanted femoral heads [2,3,4,5]. These areas were proven to be transferred metallic deposits [6,7,8,9], which are mainly localized at the equator of the heads [10]. Although studies reported the occurrence of deposits mainly on ceramic heads, metallic transfer was also detected on numerous retrieved metallic heads [11]. Independent from the material of the head, it did not matter whether the bearing partner was made of ceramic, polyethylene (PE) or metal [11,12]. 

Subsequently, the metallic deposit on the surfaces of the bearing couples leads to a change in the wetting behavior [13] and an increase in surface roughness and thus to significantly increased abrasion and wear in total hip replacement, especially with PE as a bearing partner [8,14,15]. In addition, metallic particles can be embedded in the PE surface [6]. These metallic particles can be released when the metallic deposition on the femoral heads is abraded [14,16]. Chevillotte et al. [17] report that disruption of the lubricating film between the bearing surfaces by metal transfer could be a reason for squeaking in ceramic-on-ceramic bearing couples of hip arthroplasties.

Different motion sequences result in different stress distributions in the hip joint [18]. This can lead to various wear mechanisms but also to a variety of deposition patterns. The most common pattern of deposition was described as scattered and longitudinal stripes, sometimes also randomly distributed areas, and in rare cases a completely patterned coverage of the femoral head [11]. The striped patterns are caused by line contact of the head and acetabular rim, which may occur during final repositioning while primary implantation [19,20] or due to edge loading of the hip arthroplasty [12]. Other possibilities of occurrence are during normal gait due to microseparation or unexpected activities such as stumbling [21]. Other causes for metallic deposition are mainly malpositioning, loosening or migration of implant components as well as metallic contacts during surgery [3,4,7,15,20,22,23]. The entry of wear particles from the taper connection into the articulating joint surface during prosthetic impingement can also cause metallic deposition on the head [24]. Several studies report that metallic transfer can occur with slight metallic contact [7,15,20,23,25].

The amount of total wear correlates with the extent of the deposited area [3,9,26,27]. In studies of Dorlot et al., Fredette et al. and Müller et al. [3,11,23], areas from 5 mm^2^ up to 850 mm^2^ of the transfer with a maximum height of 30 µm were determined. In some studies, energy dispersive X-ray (EDX) analysis in combination with scanning electron microscopy (SEM) was used to determine the composition of the metallic deposit on the femoral heads. In most cases, CoCr or titanium alloys were identified as transferred material. However, EDX has been performed only on small numbers of samples and only on ceramic heads, often searching for explicit materials [3,6,7,8]. 

In numerous studies, retrieved femoral heads are examined with regard to their abrasion and wear behavior. Thereby, the metallic deposition was often documented as well. However, a detailed examination of the transferred material mostly did not follow [28,29,30]. Studies that analyzed the depositions in more detail usually had a very small number of samples or examined only one head material [31,32,33]. There are just a few studies known to date that have investigated metallic deposition in a meaningful number of samples [10,11,26]. However, of these, only the study of Fredette et al. [11] included metallic heads in addition to ceramic heads. Most studies only investigate the influence of the metallic application but not its origin. So far, only the origin of the striped patterns was explained [12]. The formation of other deposition patterns such as solid or random patches is not yet clear. The knowledge of the cause of formation may help to develop new designs that might prevent the formation of the metallic deposition, such as the use of dual mobility systems to reduce dislocations [34]. Hence, the aim of our present study was to investigate the metallic transfer in terms of its composition and origin. For this purpose, retrieved femoral heads made of different materials with metallic depositions were collected (heads with a minor grade of depositions were neglected) and the composition of the transfer material was determined by means of SEM-EDX analysis. The results of the study together with the data regarding the type of bearing as well as the cause of revision in combination with the large number of samples should allow the origin of the deposited material to be determined. The results of this study are of great importance, since there is no study with a large number of samples and with such a wide variety of femoral head materials and designs that investigates the correlation of the parameters deposition material, cause of revision, type of bearing, localization and pattern of the deposition.

## 2. Materials and Methods

**Figure 1:**
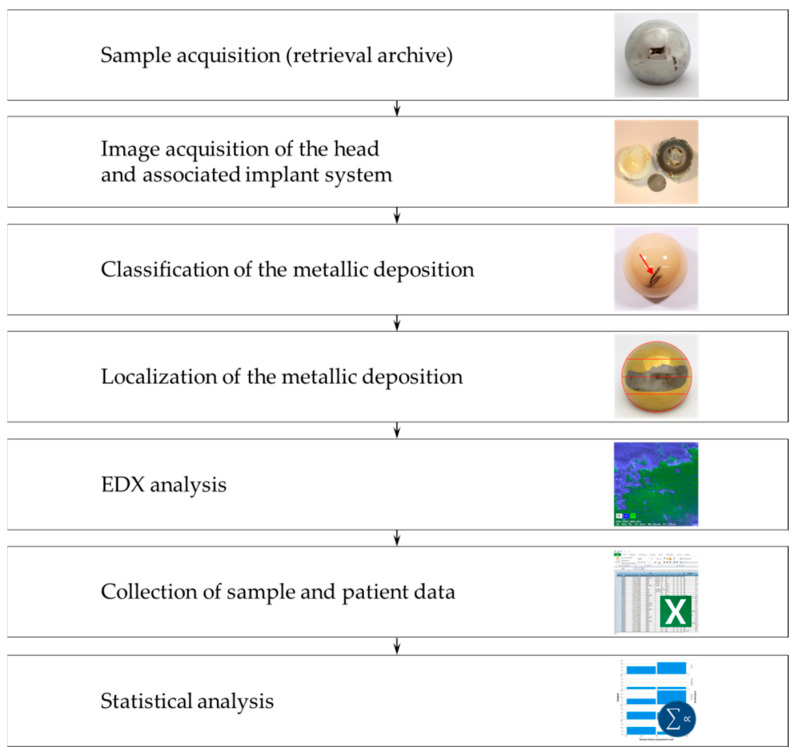
Workflow of the present study.

The workflow and methods used are summarized in Figure 1.

### 2.1. Specimens

For this study, retrieved femoral heads of various designs with metallic deposition localized on the surface were selected from the retrieval archive of our hospital. Patients signed a consent statement for the safekeeping and examination of the explants. The data protection regulations were observed. The study was approved by the local Ethics Committee (registration number A 2019-0103). The selected 98 heads consisted of five different materials. The numbers of heads per material group are listed in Table 1.

The patient data collected in reference to the selected explants were anonymized. Since many of the collected samples were not implanted at our Orthopedic Hospital, the implantation period is mostly unknown and was therefore not investigated in this study. The type of bearing materials as well as the cause for revision were considered for the evaluation of the results. After revision, all implant systems were sanitized at 95 °C. Furthermore, all femoral heads were cleaned in deionized water in an ultrasonic bath (SONOREX, BANDELIN electronic GmbH & Co., KG, Berlin, Germany) and dried with nitrogen to remove loose wear debris before examination.

An experienced and board-certified orthopedic surgeon, specially trained and approved in total joint arthroplasty at a center of excellence (Endocert-Certification Germany), was consulted for all subsequent examinations.

### 2.2. Classification and Localization of the Metallic Deposition

The deposition patterns were classified according to the study of Fredette et al. [11]. The patterns were recorded as follows: solid patch, directional scratch, longitudinal stripe, random stripe, random patch, patterned coverage and miscellaneous. The images were scored by two independent experts based on the above detailed classification. Scorings were compared and if findings differed, a third, joint assessment of the deposits was performed to obtain a consistent result.

**Figure 2:**
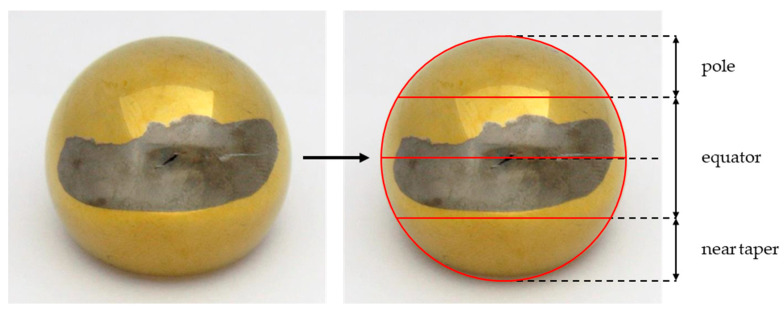
TiN-coated femoral head with metallic deposition in the equatorial area.

In order to standardize the mapping of the localization of the metallic contamination, a grid was drawn on the acquired images of the analyzed femoral heads. The grid was used to divide the head surface into the areas pole, equator and near taper. An example image of the adopted segmentation is shown in Figure 2.

During a revision, the implants are removed, cleaned and stored in single parts (unassembled) in the retrieval archive. The position in which the femoral head was mounted on the femoral stem was not documented. Therefore, an exact determination of the contact surface cannot be made for most heads.

### 2.3. EDX Analysis of Femoral Heads

To analyze the metallic coating, all femoral heads were examined a by field emission scanning electron microscope (FESEM, MERLIN^®^ VP Compact, Co., Zeiss, Oberkochen, Germany) equipped with a detector (XFlash 6/30) for EDX spectroscopy and analysis software (Quantax400, Co., Bruker, Berlin, Germany). Representative areas of the samples were analyzed and mapped to determine the elemental distribution on basis of the EDX-spectra data by the QUANTAX ESPRIT Microanalysis software (version 2.0).

**Figure 3:**
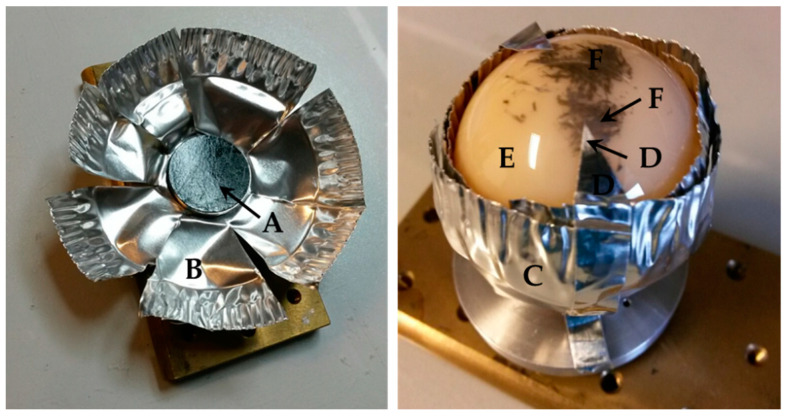
Double-sided carbon adhesive tape (A) in aluminum sample holder (B). Right: inserted ceramic femoral head in closed aluminum sample holder (C) with aluminum adhesive tape (D) on original head surface (E) and deposited area (F).

Measurements were made in the transition area from the original surface to the metallic deposition in order to show the difference between the materials. For the investigation by means of EDX, a conductive surface is necessary for the conduction of the electron beam, which is normally ensured by sputtering the sample with gold or carbon. However, in order not to contaminate the retrievals by sputtering, the ceramic heads were placed on a carbon tape in a holder made of aluminum foil and the area to be examined was made accessible with aluminum tape (see Figure 3). It was searched for many known implant and surgical tool materials (exemplary Fe, Co, Cr, Mo, V, Ti, N, Nb, Al, Zr, Y, Sr). The method was used to determine the composition and localization of the transfer material and therefore to deduce the source of the deposited material. However, an exact quantitative analysis of the material composition was not possible by EDX analysis. 

### 2.4. Statistical Analysis

Statistical analysis was performed using the IBM^®^ SPSS^®^ Statistics (version 27, IBM Corporation, New York, NY, USA). For the selected femoral heads, the absolute frequencies of the transfer materials, the deposition patterns and the cause for revision were analyzed. Cross tables with Chi-square test and Fisher’s exact test (two-sided) were used to determine associations in deposited material and deposition pattern due to the cause of revision or the localization of the depositions on the femoral head. *p*-values of <0.05 were considered significant.

## 3. Results

### 3.1. Cause of the Revision of the Examined Femoral Heads

**Figure 4:**
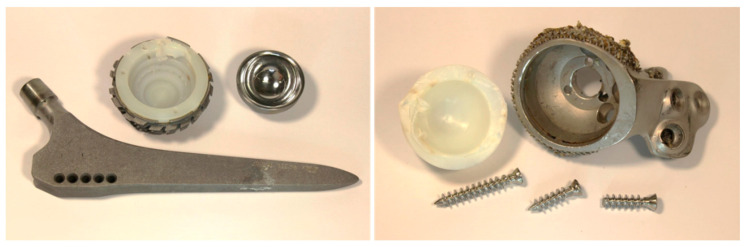
Examples of revised total hip implant systems.

In order to determine the cause of the revision, revised total hip implant systems (if available) were also examined in addition to the retrieved heads (see Figure 4).

**Figure 5:**
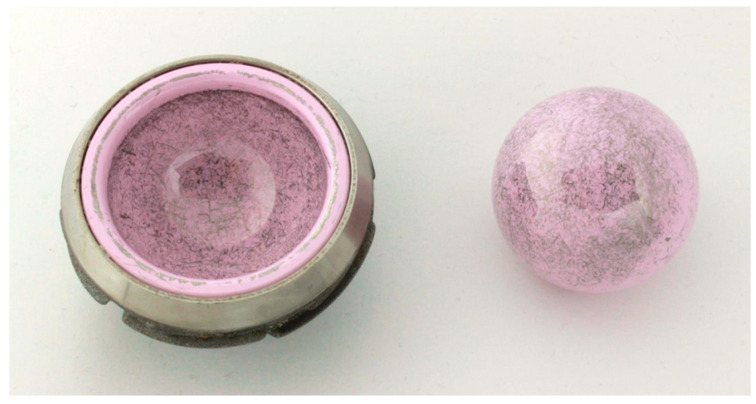
Ceramic-on-ceramic bearing couple with globally distributed metallic deposition on femoral head and insert.

The metallic deposition can also be found in part in the associated metallic or ceramic bearing surfaces (see Figure 5). However, the more noticeable contamination is found on the femoral heads.

The frequencies of the cause for revision of the retrieved heads are listed in Table 2. The most common cause for revision was wear of the polyethylene insert followed by impingement and aseptic loosening. Septic revisions and dislocations amounted to approximately 20% each. Several causes for revision may occur for the same retrieval.

### 3.2. Classification and Localization of the Metallic Deposition

Random stripes, random patches and solid patches were most prevalent on retrieved femoral heads (see Table 3). The random patches were located almost exclusively on hard-hard pairings (*p* = 0.022). A further significant correlation of the pattern or the localization of the deposition with the different bearing partners could not be found. In total, 11.2% of the heads showed global patterns distributed over the entire head.

**Figure 6:**
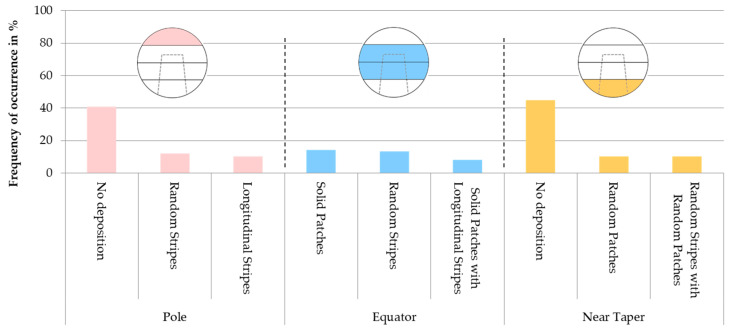
Most frequent deposition patterns on the femoral heads in the areas pole, equator and near taper.

The metallic deposition occurred most frequently in the area of the equator. In 53.1% of the heads, metallic deposits were found in the equator and pole regions without contamination of the taper-near area. However, there was no case in this study in which metallic contamination occurred exclusively in the pole area. A total of 40.8% of the heads had no metallic depositions in the pole area at all. The most prevalent deposition patterns on the pole area were random stripes (12.2%) and longitudinal stripes (10.2%). In the equatorial area, the three most recurrent deposition patterns were solid patches (14.3%), random stripes (13.3%) and a combination of solid patches and longitudinal stripes (8.2%). In the near taper area, the majority of heads also showed no deposition (44.9%). The most prevalent patterns of deposition were random patches and a combination of random stripes and random patches (10.2% each, see Figure 6).

In a statistical comparison, Fisher’s exact test (two-sided) was used to analyze whether there was an association between the metallic patterns and the cause for revision. It was found that a solid patch was detected in more than half (55%) of the femoral heads that were retrieved due to dislocation and thus a direct association cannot be excluded (*p* = 0.066).

When solid patches were present, they were always localized in the equator area (11.4% of heads with solid patches) or in the equator and adjacent area (88.6% of heads with solid patches). In nearly half of the femoral heads with solid patches, it occurred in the equator and pole region (*p* = 0.028). It was also shown that in case of dislocation, there were no random patches in 80% of the cases (*p* = 0.041) and no patterned coverage in 70% of the cases (*p* = 0.008).

A possible association exists for the occurrence of random stripes in gluteal insufficiency as well as implant fracture, which may include a fracture of the acetabular cup as well as a fracture of the surrounding bone cement (*p* = 0.087 in each case). While in both cases all heads showed random stripes, the total number of heads for these specific causes was limited with only three retrieved heads each. This makes it difficult to draw a conclusion from the results.

### 3.3. Material Analysis of Femoral Heads and EDX Analysis

**Figure 7:**
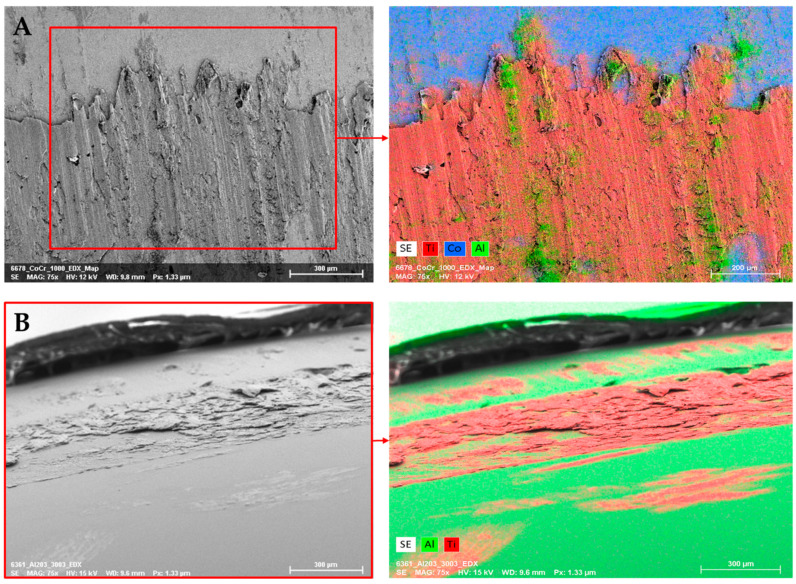
EDX mapping on retrieved femoral heads; (**A**) metallic deposit containing Ti and Al on CoCr femoral head and (**B**) metallic deposit containing Ti on alumina ceramic head.

By means of EDX analysis and mapping, individual elements could be assigned to the metallic deposition. Metallic deposit, which consisted of the same material as the original surface of the head, was only included in the analysis if it could be clearly defined as a deposition. Figure 7 shows an example of the composition of the metallic deposit based on a mapping on a CoCr and an alumina head.

The elements that were clearly detected as depositions by EDX are listed in Table 4. Titanium was detected as the most common deposition material on the retrieved femoral heads, amounting to 76.5%. Nearly half of the heads showed carbon deposition. Iron and chromium were found on approximately one third of the retrievals. Other elements were only detected with lower abundance. The bearing materials given in Table 1 were examined for correlations with the detected deposition materials. However, no correlations could be found.

Many elements, often including carbon and silicon were only detected as background noise and could not be directly defined as a deposition material. It was hypothesized that this was background noise caused by remaining material from previous revision surgeries. However, this hypothesis could not be confirmed statistically (*p* = 0.374).

In order to determine the origin of the transferred and deposited materials, it was verified whether there were statistical associations between the transfer materials and the cause for revision of the retrievals. The detailed analyses showed that some of the elements that were detected by EDX occurred more often after specific causes of revision. Only the significant associations in Chi-square test between certain elements and specific causes are described in detail. Of 34 femoral heads with depositions including Fe, 24 heads were retrieved due to PE wear (*p* = 0.035). All heads (*n* = 3) which had to be revised due to implant fracture had depositions of Fe on the head surface (*p* = 0.039). Five out of seven heads with deposition involving oxygen were retrieved due to particle disease (*p* = 0.002). Out of four heads with sulfurous deposit, three had septic loosening as the cause for revision (*p* = 0.004). It should be mentioned here that all three cases were bearing couples of alumina-on-PE. In the first case, the bacterial strains *Finegoldia magna*, *Bacillus cereus*, *Corynebacterium* species, *Staphylococcus aureus* and *Neisseria* species were detected. The second case infection was again caused by *Staphylococcus aureus*, while in the third case *Enterococcus faecalis* was the causative pathogen. For the revision cause septic (without loosening), eight out of nine heads showed carbon deposition (*p* = 0.015). One out of three heads with revision due to bone fracture (including trochanteric major contact) showed deposition of calcium (*p* = 0.031).

## 4. Discussion

Metallic depositions on retrieved femoral heads were commonly observed in total hip arthroplasty. Some experimental studies showed that the deposited material can lead to increased third body wear [6,7,14]. The appearance and extent of the transfer varies for each revision [6,11]. Slight deposits are often difficult to detect but can still have an effect on the wear behavior of the articulating surfaces. Especially on metallic heads of the same color, deposits are barely visible [12] and their existence cannot be excluded. The aim of our present study was to determine the composition and emergence of metallic deposition in order to identify indications and strategies on how metallic deposition can be avoided. For this purpose, 98 retrieved femoral heads with material transfer on the surface were analyzed. It should be mentioned that heads with only minor contamination were not included in the study. Since all of the heads were from revisions and in many cases the first implantation did not take place in our hospital, some data could not be recorded and no information about the implantation period or any previous implants could be provided. In some cases, this includes exact data of implant design used. Therefore, for femoral heads with the bearing partner PE, a subdivision into conventional and crosslinked or mixed with additives (e.g., vitamin E) was omitted. For the same reason, in many cases the exact composition of the ceramic could not be determined. For instance, in the case of the other oxide ceramics, it was unknown in some cases whether they were made of zirconia, ATZ or ZTA and whether yttrium had been added. The influence of patient age, sex, weight, or activity on the extent of transfer cannot be found in the study by Fredette et al. [11]. Furthermore, it cannot be excluded that some marks of deposition may have been caused during the implantation or revision, for example due to surgical instruments. It is also unknown which depositions may have been created by contact during storage in the explant archive. To address these limitations, the bearing partners, cups and remaining revised parts (if available) were included in the analysis. In connection with the surgery reports, this provided information about the cause of revision. During examination of the retrievals, it was found that metallic transfer was not only present on femoral heads. It was also present on the surface of the bearing partners in hard-hard bearing couples and, in some cases, also on the associated retrieved implant systems.

A study of Brand et al. [19] reported that mainly stripe-shaped patterns were seen on retrieved femoral heads, while predominantly smear-typed patterns were seen on retrieved acetabular cups. In the study of Fredette et al. [11], the transfer patterns on the femoral heads were classified for the first time. As this classification scheme was also used in this study, a similar distribution of the deposition patterns was observed as compared to the one described by Fredette et al. [11]. A significant association was found between the occurrence of random patches and ceramic-ceramic bearing couples. One reason for this is that particles were ground by the harder ceramic after entering the bearing surfaces and were distributed in the entire joint space during articulation. This could also have resulted in patterned coverage as it is shown in Figure 4. No further association of the occurring transfer patterns and the type of bearing couple was found. Contrary to the study of Fredette et al. [11] where the transfer was mainly located in the upper hemisphere (equivalent to pole), the transfer patterns in our present study were mainly located in the equatorial region, but especially in the transitional region from the equator to the pole region. A distinctive pattern often found in this region was the solid patch. This pattern was associated with a recurrent dislocation. The dislocation patch could be caused by scraping the head on the rim of the metallic acetabular cup and distributing the generated metallic particles on the articulating surface after repositioning, which is also described in the case report of Patten et al. [33]. There were also significantly more occurrences of random stripes in revisions due to gluteal insufficiency and implant fracture. The pattern could occur in these unstable situations due to the linear contact of the femoral head with the rim of the acetabular cup. A study by Walter et al. [12] reported that striped transfer could be caused by edge loading. However, due to the wide variety of causes there were often only a small number of samples available per group in this study to test for associations.

Since the articulating joint is surrounded by a joint capsule, it is likely that the transferred material originates from wear particles of the implant as well as the bone and the surrounding tissue. The main components of metallic acetabular cups and stems are Ti alloys (mainly Ti-6Al-4V) and CoCrMo alloys. Thereby, Ti alloys are the softer material and are often associated with tissue discoloration and the release of wear particles [35]. Additionally, in this study, as well as in the study of Tikekar et al. [31], titanium is most frequently found transfer material on the retrieved femoral heads. The second most common material was carbon, which probably originates from bone wear particles that may be generated during the implantation process or by micromotions between bone and implant. Sources for the other detected elements could be ceramic particles from ceramic heads, in which mixed ceramics of Al_2_O_3_ and ZrO_2_ with added yttrium are often used [36]. Wear particles from bone cement would also be conceivable. This consists primarily of polymethyl-methacrylate (C_5_O_2_H_8_), but may also contain components of ZrO_2_, BaSO_4_ (barium sulfate), Na, P, Ca, Si and Cl. The elements Na, P, Ca and Cl are also components of the blood plasma and can diffuse into the bone cement over time [37]. Wear particles from surgical tools, which were introduced during implantation or revision, cannot be excluded. Residues of previous implants in case of multiple revisions can remain in the body for a long time and could be an explanation for the background noise that was observed on some heads during EDX analysis. It is assumed that, especially in the case of pronounced depositions, several transfer layers are present, between which tissue and synovial residues have become embedded. In the present study, iron was found to be a transfer material in total hip revisions associated with polyethylene wear. This represents delamination and also decentralization, in which the femoral head ploughs into the liner out of the center of rotation. This enlarges the artificial joint gap and enables the generated wear particles to migrate more easily within the articulating surfaces. In the case of heavily abraded bearings, the femoral head can also come into contact with the outer cup as well as additional fixation screws. These screws are often made of stainless steel and could be a reason for the transferred Fe. Furthermore, the significant occurrence of Fe as a deposit after implant fractures may originate from contact with additional fixating implants. In case of revision due to wear particle disease, the deposit of O was significantly detected. Since only elements can be detected by EDX, but not element compounds, the origin cannot be clarified. A conceivable cause may be the formation of free oxygen radicals, which often occur in wear particle disease due to their biological response [38,39,40]. Oxidized metal particles are also a possible cause. An advanced analysis method represents X-ray photoelectron spectroscopy (XPS), which can provide information about the elemental compounds on the surface transfer. This could allow for a more precise delimitation of the origin of the transferred material. 

In the case of septic loosening, a significant occurrence of sulfur was found in the transferred materials on the femoral heads. A possible source could be in the biofilm formed by colonizing bacteria [41]. The bacterial pathogens detected in the revisions *Staphylococcus aureus* and *Enterococcus faecalis* are among the most common pathogens in septic revisions [42]. Some studies showed that sulfide is an important component of the microbial sulfur cycle and *S. aureus* can use hydrogen sulfide to protect against cationizing molecules from antibiotics [43,44,45]. *E. faecalis* is also known to produce sulfur-containing radicals to reduce oxidative stress [46]. Alternatively, the sulfur could originate from defensins. These small, disulfide-rich, cationic peptides are known for their antimicrobial activity and represent the first defense of the innate immune system against Gram-positive and Gram-negative bacteria, fungi, viruses and parasitic protozoa [47]. In septic loosening an abundance of these cysteine-rich peptides in the local environment of the infected implant is therefore highly likely. Another source of the sulfur could be bone cement spacers that were used as a temporary placeholder in case of implant-associated infections [37]. Carbon was another detected element that was significantly higher on femoral heads revised due to sepsis. As carbon is the main element in all organic compounds including biomolecules, the inflammatory reaction and tissue destruction in sepsis might explain why carbon is more abundant in septic revision. On heads revised due to bone fractures, the significantly detected calcium can be explained by contact with bone fragments. Due to smaller group size in the associations, these observations should be confirmed in further studies. In addition, future studies should include femoral heads with slight transfer, which were excluded from present study. In future studies, accurate visual assessment during revision will also help to distinguish existing deposits from intraoperatively generated metallic transfer.

The limitations of the study are summarized below. Due to the high variability of head materials and deposition forms, the group sizes differ remarkably. For example, a very large number of heads made of alumina were examined (*n* = 28), but only comparatively few coated metallic heads (*n* = 6). A critical limiting factor is the patient data. Because the primary surgeries often took place in other clinics, the implantation period and some other data could not be collected and thus could not be included in the correlation. After revision, specimens must be initially stored for one year before examination, as the patient is allowed to recall the retrieval within this time. During storage, additional metallic deposition by the enclosed implant system cannot be excluded. Therefore, heads with very slight deposition were excluded in the sample acquisition. In addition, only single elements could be detected by EDX analysis. The advantage of the EDX analysis is the non-destructive measurement. Nevertheless, a detailed investigation of the chemical composition is desirable. Furthermore, it is assumed that the metallic deposit is formed layer by layer on the surface. Therefore, an erosive analysis to investigate the individual layers of the metallic deposition would be of interest.

## 5. Conclusions

Material deposits are not only found on femoral ceramic heads. They can also occur on metallic heads and on the surface of the rest of the revised implants parts, such as acetabular cups or hip tapers. Our study showed that titanium is the most commonly transferred material. However, the deposition materials were not limited to metallic elements, and non-metallic materials such as carbon or sulfur were also transferred. Some possible sources of the deposition materials could be identified; for example, random patterns due to implant system contact in unstable joint situations. It was further shown that the deposition pattern solid patch results from dislocations of the femoral head. Random patches were identified as a typical pattern on ceramic-on-ceramic bearing couples. In general, depositions on bearing surfaces should be avoided, as they lead to an introduction of third-body particles and increased wear. Further long-term studies, with complete information on the implantation period and photo documentation during revision, are recommended. An examination of the metallic deposition with XPS to determine the complete elemental composition can provide more precise information on the origin of the deposition. In order to better comprehend the origin and formation of the metallic deposition and to better assess its influence on abrasive wear, the use of simulation models would be a promising option [21]. Investigation of the formation processes by means of multi-body simulation can also provide a lot of information in the future.

## Figures and Tables

**Table 1:** Selection of femoral heads with metallic depositions on original surface and associated bearing partners (MoM = metal on metal, MoPE = metal on PE, CoC = ceramic on ceramic, CoPE = ceramic on PE, unknown = unknown bearing partner).

Femoral Head Material	Bearing Partners	Elements Included in Original Surface	Example Image
Metal *n* = 28	MoM (*n* = 8) MoPE (*n* = 19) Unknown (*n* = 1)	Co, Cr, Mo	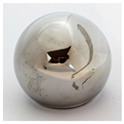
Coated Metal TiN/TiNbN *n* = 6	MoM (*n* = 6)	Ti, N, Nb (Co, Cr, Mo beneath coating)	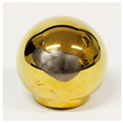
Alumina Ceramic *n* = 28	CoC (*n* = 1) CoPE (*n* = 26) Unknown (*n* = 1)	Al, O	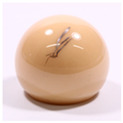
ZTA Ceramic (Zirconia-Toughened Alumina) *n* = 21	CoC (*n* = 1) CoPE (*n* = 20)	Al, O, Zr, Y	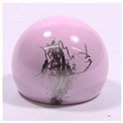
Other oxide ceramics ATZ/ZTA/ZrO (Zirconia-Toughened Alumina) *n* = 15	CoC (*n* = 1) CoPE (*n* = 13) Unknown (*n* = 1)	Al, Zr, O, Y	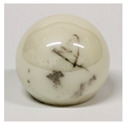

**Table 2:** Occurring causes for revision of the examined retrievals.

Cause for Revision	Frequency of Occurrence in %	Absolute Number (Total *n* = 98)
Polyethylene wear (includes decentralization, delamination, linear wear)	43.9	43
Impingement	40.8	40
Aseptic loosening (with 25.5% global loosening, 11.2% cup loosening, 1% stem loosening)	37.7	37
Dislocation (includes single and multiple dislocation)	20.4	20
Particle disease (due to PE and metallic wear particles)	17.3	17
Septic loosening	11.2	11
Septic, without loosening (includes DAIR ^1^)	8.2	9
Implant migration	5.1	5
Gluteal insufficiency	3.1	3
Osteolysis	3.1	3
Bone fracture	3.1	3
Implant failure	3.1	3
Subluxation	2.0	2

^1^ DAIR = debridement, antibiotics and implant retention.

**Table 3:** Occurring deposition patterns on femoral heads.

Deposition Patterns on Femoral Heads According to Fredette et al. [11]	Frequency of Occurrence in %	Absolute Number (Total *n* = 98)	Exemplary Deposition Pattern on Heads of This Study
Random Stripes	44.9	44	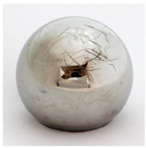
Random Patches	41.8	41	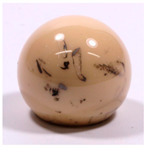
Solid Patch	35.7	35	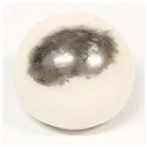
Longitudinal Stripe	27.6	27	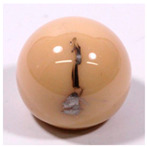
Directional Scratches	20.4	20	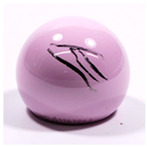
Patterned Coverage	11.2	11	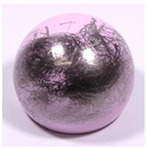
Miscellaneous	2.0	2	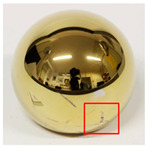

**Table 4:** Detected deposition materials on femoral heads.

Element	Frequency of Occurrence on Femoral Heads %	Absolute Number (Total *n* = 98)
Titan (Ti)	76.5	75
Carbon (C)	49.0	48
Iron (Fe)	34.7	34
Chromium (Cr)	31.6	31
Niobium (Nb)	23.5	23
Cobalt (Co)	16.3	16
Aluminum (Al)	12.2	12
Vanadium (V)	9.2	9
Nickel (Ni)	8.2	8
Oxygen (O)	7.1	7
Nitrogen (N)	6.1	6
Silicon (Si)	5.1	5
Molybdenum (Mo)	4.1	4
Sulfur (S)	4.1	4
Magnesium (Mg)	3.1	3
Phosphorus (P)	2.0	2
Copper (Cu)	1.0	1
Zircon (Zr)	1.0	1
Calcium (Ca)	1.0	1

## Data Availability

Not applicable.
